# Coronavirus HKU1 Infection in the United States

**DOI:** 10.3201/eid1205.051316

**Published:** 2006-05

**Authors:** Frank Esper, Carla Weibel, David Ferguson, Marie L. Landry, Jeffrey S. Kahn

**Affiliations:** * Case Western Reserve University School of Medicine, Cleveland, Ohio, USA;; † Yale University School of Medicine, New Haven, Connecticut, USA

**Keywords:** Coronaviruses, Coronavirus HKU1, HCoV-HKU1, clinical features, respiratory infections

## Abstract

In 2005, a new human coronavirus, HCoV-HKU1, was identified in Hong Kong. We screened respiratory specimens collected from December 16, 2001, to December 15, 2002, from children <5 years of age who tested negative for respiratory syncytial virus, parainfluenza viruses, influenza virus, and adenovirus for HCoV-HKU1 by reverse transcription–polymerase chain reaction. Overall, 1,048 respiratory specimens from 851 children were tested, and 9 HCoV-HKU1–positive children (1%) were identified, 2 of whom had 2 positive specimens. Children who had HCoV-HKU1 infection had evidence of either upper or lower respiratory tract infection or both. Two patients had disease beyond the respiratory tract. HCoV-HKU1 was identified from December 2001 to February 2002. Sequence analyses suggest that a single strain was circulating. HCoV-HKU1 is therefore likely circulating in the United States and is associated with upper and lower respiratory tract disease.

## Abstract 2

Virus is associated with respiratory tract disease in children <5 years of age.

Lower respiratory tract disease accounts for ≈4 million deaths annually worldwide (*1*). Viruses such as influenza virus, respiratory syncytial virus (RSV), and parainfluenza viruses are responsible for much of this respiratory tract infection. However, in a substantial proportion of respiratory tract disease, no pathogen is identified (*2*).

Coronaviruses (CoV) infect a wide variety of mammals and birds, causing disease of the respiratory tract, gastrointestinal tract, and central nervous system. These viruses may be transmitted from species to species (*3*). In humans, CoV have been associated with community-acquired upper respiratory tract infections (*4*). Human CoV (HCoV) have also been implicated in outbreaks of diarrhea as well as in demyelinating disorders of the central nervous system, though these data are controversial (*5**,**6*). The study and identification of HCoV have been hampered by the difficulty in propagating these viruses in vitro.

The identification of the severe acute respiratory syndrome–associated CoV in 2003 sparked renewed interest in the study of HCoV (*7*), and 4 previously unidentified HCoV have subsequently been discovered. HCoV-NL63, HCoV-NL, and the New Haven coronavirus (HCoV-NH) are closely related group I CoV and likely represent strains of the same species of virus (*8**–**10*). HCoV-NL63 and HCoV-NL were originally identified by cell culture techniques, while HCoV-NH was discovered by using broadly reactive CoV molecular probes. These related viruses were identified in both children and adults with respiratory tract disease. HCoV-NH was found in 8.8% of children <5 years of age whose specimens originally tested negative for RSV, influenza virus, parainfluenza viruses, and adenoviruses (*10*). Furthermore, these newly discovered viruses may be the cause of disease beyond the respiratory tract. In a case-control study, HCoV-NH was found to be associated with Kawasaki disease (*11*), although these data are controversial (*12**,**13*).

In 2005, Woo et al. reported a novel group II CoV, designated HCoV-HKU1, from a 71-year-old man with pneumonia (*14*) who had recently returned to Hong Kong from the Shenzhen, China. As in the discovery of HCoV-NH (*10*), this virus was detected with molecular probes. Although growth of HCoV-HKU1 in multiple cell lines was unsuccessful, the complete genomic sequence was obtained. Phylogenetic analysis showed that this new group II CoV is most closely related to the mouse hepatitis virus and is distinct from HCoV-OC43, the only other known group II HCoV. Screening of 400 nasopharyngeal aspirates by reverse transcription–polymerase chain reaction (RT-PCR) with HCoV-HKU1–specific primers showed 1 other HCoV-HKU1 isolate from a 35-year-old woman with pneumonia. After the original report, HCoV-HKU1 was identified in 10 patients in northern Australia (*15*). Respiratory samples were collected between May and August (winter in Australia) and screened by RT-PCR with both nonspecific CoV and specific HKU1 primers. Most HCoV-HKU1–positive samples originated from children in the later winter months. However, the seasonal and geographic distribution of this virus is still unclear. To address these issues, we sought to determine whether HCoV-HKU1 circulated in New Haven, Connecticut, and to define clinical characteristics associated with HCoV-HKU1 infection in infants and children.

## Methods

### Clinical Specimens

Nasopharyngeal swabs and aspirates submitted to the clinical virology laboratory at Yale–New Haven Hospital from December 16, 2001, to December 15, 2002, for respiratory virus diagnosis were initially tested for RSV, parainfluenza viruses (types 1–3), influenza A and B viruses, and adenovirus by direct immunofluorescence assay. Respiratory specimens were screened for human metapneumovirus (*16*) and HCoV-NH (*10*) by RT-PCR. Specimens originated from the emergency department, inpatient wards, intensive care units, and the hospital-affiliated primary care outpatient clinic and were submitted at the discretion of the medical teams. Clinical specimens from children <5 years of age that tested negative by direct immunofluorescence assay were tested for HCoV-HKU1 as described below. Collection of specimens and clinical data was approved by the Yale University Human Investigation Committee and compliant with Health Insurance Portability and Accountability Act regulations.

### RT-PCR Screening

RNA from each respiratory specimen was extracted with the QIAamp Viral RNA Mini Kit (Qiagen, Valencia, CA, USA), according to the manufacturer's protocol. Random hexamer primers synthesized by the oligonucleotide laboratory, Department of Pathology, Yale University School of Medicine, were used to create a cDNA library for each specimen. Reverse transcription reactions were performed with MuMLV RT (New England Biolabs, Beverly, MA, USA), according to the manufacturer's specifications. Each cDNA was subsequently screened for the presence of HCoV-HKU1 by polymerase chain reaction with HotStar Taq polymerase (Qiagen), according to the manufacturer's specification. Primers used to screen respiratory specimens were identical to those described by Woo et al. (*14*). The forward primer, 5´ GGTTGGGATTATCCTAAATGTGA, and reverse primer, 5´ CCATCATCACTCAAAATCATCATA, produce an amplicon that corresponds to nucleotides 15409–15848 of the HCoV-HKU1 replicase 1B gene (GenBank accession no. AY597011) and yields an amplicon of 439 bp. Amplification cycles were as follows: 95°C for 15 min; followed by 40 cycles of 94°C for 1 min, 55°C for 1 min, and 72°C for 1 min; and completed with a final extension cycle of 72°C for 10 min. Each set of reverse transcription and polymerase chain reactions contained appropriate negative controls. Sequencing was performed on an Applied Biosystems 3730 XL DNA Analyzer (Foster City, CA, USA) at the W.M. Keck Biotechnology Resource Lab, Yale University School of Medicine.

### Clinical Data

Medical records of all HCoV-HKU1–positive children were reviewed. Demographic data, history of illness, and results of clinical examination and laboratory studies were recorded on a standard collection form. The Yale University Human Investigation Committee approved collection of specimens and clinical data.

## Results

From December 16, 2001, to December 15, 2002, 1,048 respiratory specimens from 851 children were tested by RT-PCR for HCoV-HKU1. Specimens from 9 of these children (1%) tested positive for HCoV-HKU1. Specimens from these children tested negative for RSV, parainfluenza viruses (types 1–3), influenza A and B viruses, and adenovirus by direct immunofluorescence assay as well as human metapneumovirus and HCoV-NH by RT-PCR. Two children had 2 specimens that tested positive for HCoV-HKU1. For each of these 2 children, the positive specimens were collected <10 days apart. Children whose specimens tested positive for HCoV-HKU1 infection had clinical evidence of either upper or lower respiratory tract infection or both (Table). The most common clinical findings were rhinorrhea (100%), cough (67%), fever (67%), and abnormal breath sounds on auscultation (44%). Hypoxia (oxygen saturation of <90%) was observed in only 1 patient. Chest radiographs were obtained for 4 patients, all of whom had abnormal findings that included peribronchial cuffing, atelectasis, hyperinflation, or infiltrates. One patient (patient 3) had respiratory decompensation requiring ventilatory support and was admitted to the pediatric intensive care unit. This patient had no history of underlying illness, had not been premature, and was 1 month of age at the time of specimen collection.

**Table:** Clinical manifestations associated with human coronavirus HKU1 infection*

Patient no. (sex)	Age (mo)	Specimen collection date	Length of hospitalization (d)	Diagnosis	Underlying illness	Signs/symptoms	Chest radiographic findings
1 (M)	4	Dec 18	1	New onset seizures	RAD	Rhinorrhea, cough	Not obtained
2 (M)	12	Dec 20	4	Pneumonia	Cystic fibrosis	Fever, rhinorrhea, cough, wheezing, rhonchi, retractions, rash	Infiltrates
3 (F)†‡	1	Dec 22, 31	9	Pneumonia	None	Fever, hypoxia, rhinorrhea, cough, wheezing, rhonchi, retractions, nasal flaring, apnea	Hyperinflation, infiltrates, peribronchial cuffing, atelectasis
4 (M)	2	Jan 5	NH	Fever	None	Fever, rhinorrhea	Not obtained
5 (F)†	20	Jan 14, 16	2	ALTE	None	Fever, rhinorrhea, cough	Infiltrates, atelectasis
6 (F)	3	Jan 16	NH	Bronchiolitis	Prematurity (34 weeks)	Rhinorrhea, cough, wheezing, rhonchi	Peribronchial cuffing
7 (M)	13	Jan 19	10	Hepatitis	Biliary atresia, liver transplantation	Rhinorrhea, rhonchi, abnormal LFT results	Not obtained
8 (F)	16	Jan 19	1	Fever	Sickle cell anemia	Fever, rhinorrhea	Not obtained
9 (F)	13	Feb 1	NH	Fever	None	Fever, rhinorrhea, cough	Not obtained

*M, male; F, female; RAD, reactive airway disease; NH, not hospitalized; ALTE, apparent life-threatening event; LFT, liver function tests (aspartate aminotransferase 238 U/mL, alanine aminotransferase 373 U/mL, alkaline phosphatase 406 U/mL, bilirubin [total/direct] 0.15/0.05 mg/dL). †Two respiratory specimens tested positive for human coronavirus HKU1. ‡Patient required mechanical ventilation and admission to the pediatric intensive care unit.

Two patients had evidence of disease beyond the respiratory tract. One patient (patient 1) was hospitalized for new-onset seizures. Workup for a central nervous system infection, including a lumbar puncture and head magnetic resonance imaging, was unrevealing. Although a febrile seizure remains a possible diagnosis, no evidence of fever was reported by the mother or noted during the hospital stay. A second patient (patient 7) was hospitalized with hepatitis. This patient had undergone liver transplantation 3 months before admission. Immunosuppressive medications included tacrolimus and prednisolone. The patient was also receiving ganciclovir for cytomegalovirus prophylaxis. The onset of abnormal liver enzyme levels occurred several days after the onset of respiratory symptoms and after collection of the respiratory specimen that tested positive for HCoV-HKU1. No evidence of abnormal liver function was detected (both prothrombin time and partial thromboplastin time were within normal ranges). Serologic assays for hepatitis viruses A, B, and C were negative. A liver biopsy specimen did not show evidence of rejection. Levels of the serum liver enzymes slowly decreased during hospitalization. No interventions (e.g., changes in immunosuppressive therapy) were performed.

All HCoV-HKU1 infections occurred during a 7-week period from December 2001 to February 2002 (Figure). HCoV-HKU1–positive samples accounted for 5% of samples screened during that period. No HCoV-HKU1–positive isolates were detected in specimens collected in the remainder of the study period.

**Figure:**
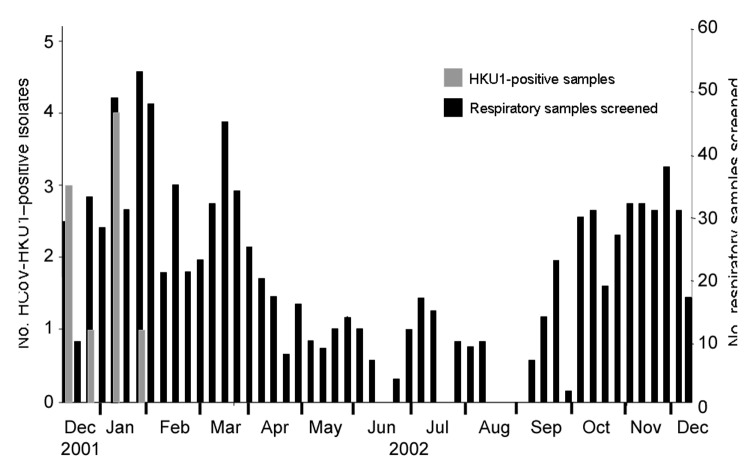
Weekly distribution of human coronavirus (HCoV)-HKU1 infection in children <5 years of age, December 16, 2001, to December 15, 2002, New Haven, Connecticut. The weekly distributions of HCoV-HKU1 isolates are shown as gray bars (left axis). The total number of samples collected by week are indicated by black bars (right axis).

The RT-PCR amplicon from each positive specimen was sequenced. Nucleotide and amino acid identity between replicase 1B region of the original HCoV-HKU1 isolate and the New Haven isolates were both >95%. Rare polymorphisms (<1% of sequence) were noted in the HCoV-HKU1 sequences of the New Haven isolates (data not shown), which suggests that a single strain was circulating in the community during the study period.

## Discussion

We report the first identification of HCoV-HKU1 in the Western Hemisphere. These findings suggest that HCoV-HKU1 may have a worldwide distribution. We detected this coronavirus in 1% of children screened. All HCoV-HKU1–positive samples tested negative for RSV, influenza virus, parainfluenza viruses, adenoviruses, HCoV-NH, and human metapneumovirus. Our laboratory did not have access to materials from Hong Kong; therefore, the results cannot represent laboratory contamination from material obtained elsewhere. The percentage of positive specimens was similar to that described by Woo et al. (1 [0.25%] of 400) (*14*) and Sloots et al. (10 [3.1%] of 324) (*15*), which suggests that infection with HCoV-HKU1 may be uncommon or that the virus has properties that decreases the likelihood of detection, such as a brief period of viral shedding. Our study, the study by Sloots et al., and the original study by Woo et al. screened respiratory specimens submitted to a diagnostic laboratory. Therefore, HKU1 may be a common virus that causes symptomatic disease in only a relatively small percentage of infected persons. All HCoV-HKU1–positive specimens were collected from December 2001 to February 2002, which implies a winter distribution. The study by Sloots et al. also detected HCoV-HKU1 predominantly in the winter, although only respiratory samples submitted during winter months were screened. Whether the seasonal distribution of HCoV-HKU1 varies from year to year is not known.

Similar to the patients described by Woo et al., several HCoV-HKU1–positive patients had evidence of lower respiratory tract involvement (2 patients with pneumonia and 1 patient with bronchiolitis). Two of these patients had underlying illness. However, most patients identified in our study had only mild upper respiratory tract symptoms. Most HCoV-HKU1 infections in children, similar to other common HCoV infections, likely result in mild disease (*4*). The Australian study did not perform a detailed clinical review of HCoV-HKU1–positive patients, but the authors note that symptoms are consistent with those of acute respiratory tract illness (*15*).The severity of disease caused by SARS-CoV in children was also relatively mild for reasons that are not yet understood (*17*). Underlying illness and preexisting lung disease may predispose to a more severe clinical course.

Evidence of hepatitis in 1 child who tested positive for HCoV-HKU1 is an intriguing finding. HCoV-HKU1 is most closely related to the murine hepatitis virus, a virus that causes hepatitis as well as demyelinating disease in mice (*18*). Because of this patient's medical history (liver transplantation) and compromised immune status, many potential causes of hepatitis exist, though serologic assays and liver biopsy findings were unrevealing. Several reports have found coronavirus-like particles in stool of persons with gastrointestinal disease (*19*), which suggests that these viruses, like coronaviruses of animals, can cause disease of the gastrointestinal tract. Future studies will be needed to determine whether HCoV-HKU1, or other common human coronaviruses, play a role in liver disease.

Our study had several shortcomings. We limited our screening to respiratory specimens that were collected at the discretion of the medical team, we did not include a control group of asymptomatic children, and serum samples were not available for serologic assays. Nonetheless, our findings show that HCoV-HKU1 is circulating in New Haven, Connecticut, and is associated with both upper and lower respiratory tract disease and perhaps extrapulmonary disease.

The genetic variability of HCoV-HKU1 is unknown. The study by Sloots et al. suggests 2 genotypes when comparing the Australian isolates to the prototype Hong Kong strain (*15*). If multiple genotypes exist, they may not all be detected with the primer set used. This limitation would result in an underestimation of this virus in our study. However, the region of the replicase 1B gene targeted by the primers used (*14*) is highly conserved among other coronaviruses, and our screening was unlikely to have lacked sensitivity for that reason. Also, only rare polymorphisms were detected on the sequence analysis of the 9 individual isolates, which suggests that this region is highly conserved. However, to establish the true prevalence of HKU1, use of primers with known specificity and sensitivity for HCoV-HKU1 will be critical.

In conclusion, we show that HCoV-HKU1 circulates in the United States, and the strain identified in New Haven is similar to the original strain described from Hong Kong. Whether this newly recognized pathogen is responsible for a substantial proportion of respiratory tract disease in children remains to be determined. Future studies are required to determine the epidemiologic features and clinical spectrum of this newly recognized pathogen.
